# Rheological Considerations of Pharmaceutical Formulations: Focus on Viscoelasticity

**DOI:** 10.3390/gels9060469

**Published:** 2023-06-07

**Authors:** Lívia Budai, Marianna Budai, Zsófia Edit Fülöpné Pápay, Zsófia Vilimi, István Antal

**Affiliations:** Department of Pharmaceutics, Semmelweis University, 1092 Budapest, Hungary; budai.livia@semmelweis.hu (L.B.); budaimarianna@gmail.com (M.B.); papay.zsofia@pharma.semmelweis-univ.hu (Z.E.F.P.); vilimi.zsofia@semmelweis.hu (Z.V.)

**Keywords:** viscoelastic gel, oscillatory rheology, viscosity, elastic and viscous moduli, biomedicine

## Abstract

Controlling rheological properties offers the opportunity to gain insight into the physical characteristics, structure, stability and drug release rate of formulations. To better understand the physical properties of hydrogels, not only rotational but also oscillatory experiments should be performed. Viscoelastic properties, including elastic and viscous properties, are measured using oscillatory rheology. The gel strength and elasticity of hydrogels are of great importance for pharmaceutical development as the application of viscoelastic preparations has considerably expanded in recent decades. Viscosupplementation, ophthalmic surgery and tissue engineering are just a few examples from the wide range of possible applications of viscoelastic hydrogels. Hyaluronic acid, alginate, gellan gum, pectin and chitosan are remarkable representatives of gelling agents that attract great attention applied in biomedical fields. This review provides a brief summary of rheological properties, highlighting the viscoelasticity of hydrogels with great potential in biomedicine.

## 1. Introduction

Viscoelastic hydrogels have the unique property of changing from a viscous character to an elastic one and vice versa [1]. Synovial or tear fluids in the human body have viscoelastic characteristics, which makes them multipotent liquids. Synovial fluids in the knee joint facilitate the movement of adjacent bones and act as a fluid-like, viscous lubricant in the joint if no stress is applied. However, under stress conditions, the knee joint requires the maintenance of the pain-free condition and avoidance of bone collisions and fractures. Therefore, the application of force to the knee joint implicates the smart alteration of the rheological behavior of joint fluid, resulting in a strengthened and more resistant elastic synovial fluid from the viscous or liquid-like joint fluid [2].

Similarly, tear fluid moisturizes the corneal surface and prevents it from drying out at rest. In contrast, the application of stress, manifested as blinking, makes tear fluid more elastic rather than viscous, preventing tears from being removed from the surface of the cornea [3,4].

**Figure 1:**
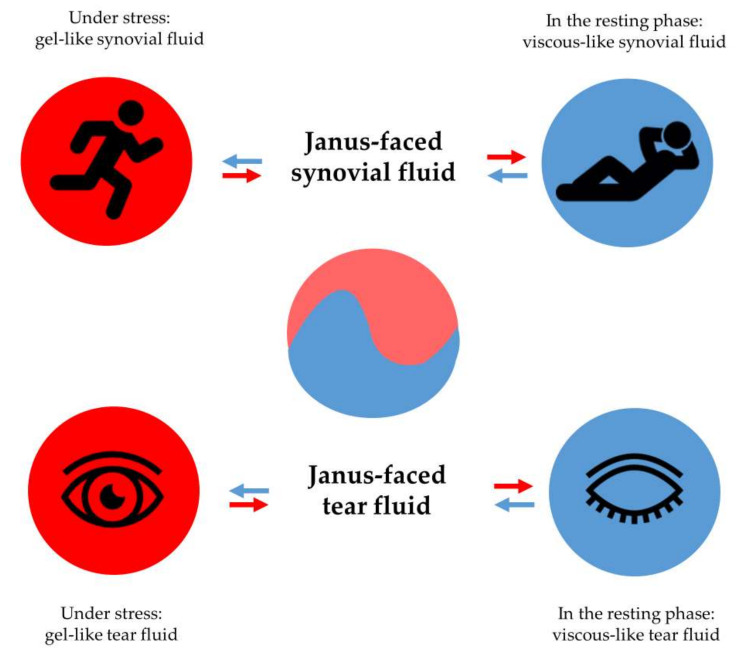
Stress-controlled behavior of viscoelastic body fluids: synovial fluid and tear fluid.

The Janus-faced property of the body fluids mentioned guarantees the perfect behavior of biological fluids under all circumstances. At rest, without pronounced stress, the viscous property of body fluids dominates. In contrast, under stress, the elastic property exceeds the viscous one (Figure 1) [5].

Oscillatory rheological experiments can describe the viscoelastic characteristic by using low frequencies to simulate conditions at rest and high frequencies that mimic conditions under stress. Furthermore, oscillatory measurements are sensitive enough to identify differences between the entanglement rate of rheological bodies and can measure the sample parameters without destroying the physical structure of the gels [6,7].

**Figure 2:**
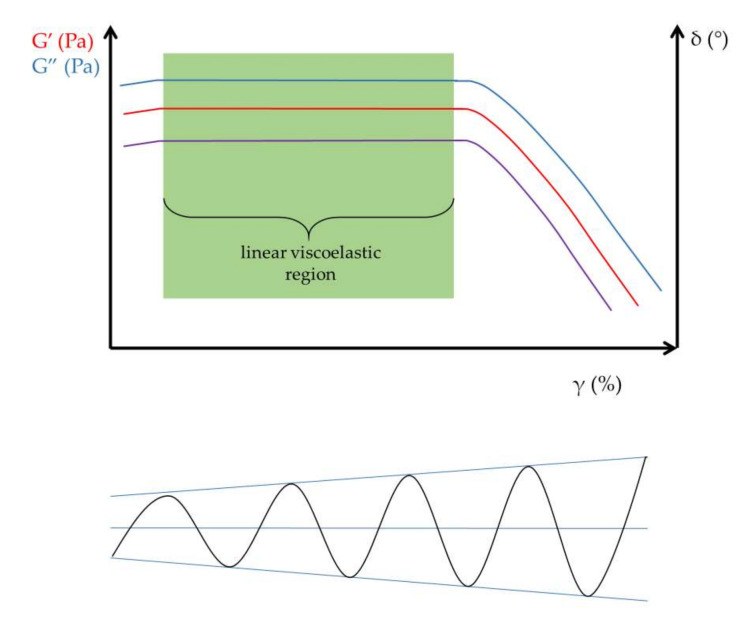
Determination of the linear viscoelastic region (LVR). The purple line demonstrates the phase angle (δ).

To determine the viscoelastic character, it is useful to perform amplitude sweep tests before measuring the frequency-dependent behavior of samples. Amplitude sweep tests are capable of the identification of the linear viscoelastic region (LVR), the range within which reproducible oscillatory tests can be performed without structural degradation of the hydrogels (Figure 2). The amplitude sweep test, as the name suggests, measures the elastic or storage modulus (G′) and viscous or loss modulus (G″) as a function of applied strain (γ).

**Figure 3:**
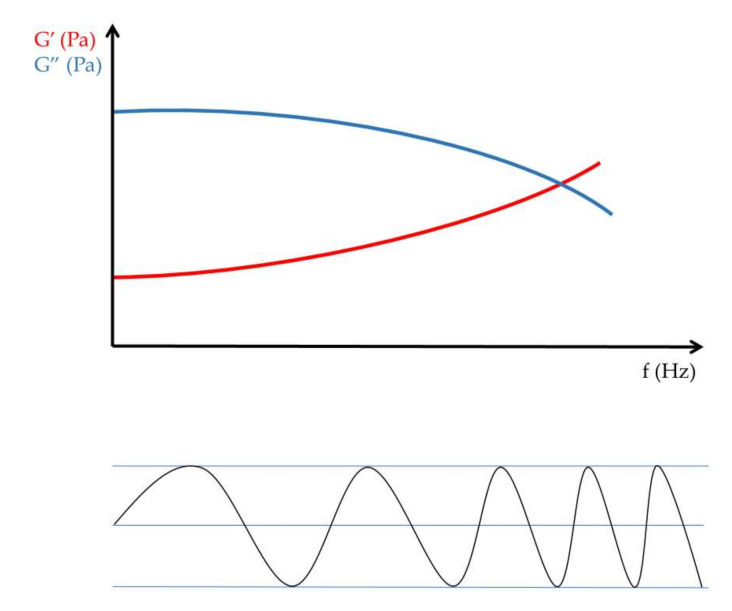
Frequency sweep measurement.

Frequency sweep tests measured in the LVR are used to scan the elastic modulus and the viscous modulus within the selected frequency range (Figure 3).

**Figure 4:**
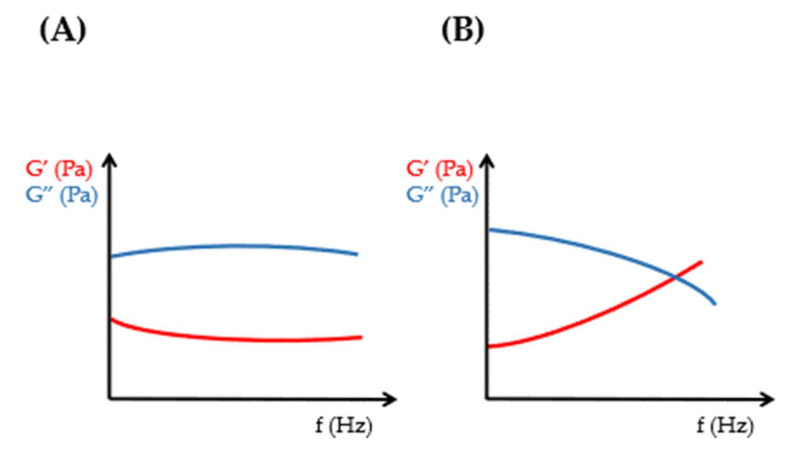
Oscillational rheology of (**A**) non-viscoelastic and (**B**) viscoelastic samples.

The value and ratio of elastic and viscous moduli are informative for the evaluation of rheological properties and further comparison of viscoelastic formulations [8,9,10,11]. The cross-over point of frequency sweep tests indicates the sol–gel transition of viscoelastic samples (Figure 4).

**Figure 5:**
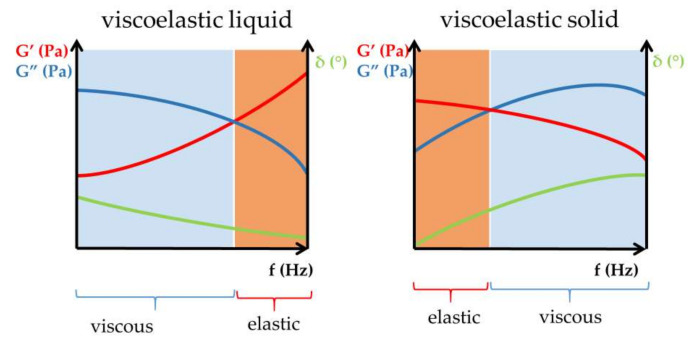
Viscous and elastic character of viscoelastic materials.

For viscoelastic fluids, the viscous modulus dominates in the lower frequency range of frequency sweep tests, while in the higher frequency range—above the cross-over point—the elastic modulus exceeds the viscous one [12]. The described behavior of the elastic and viscous moduli in different frequency ranges of frequency sweep tests represents the viscoelastic attitude of liquid-like viscoelastic hydrogels. In contrast, for solid-like viscoelastic materials, the elastic modulus dominates at low frequencies below the cross-over point of the viscoelastic transition, and the viscous modulus exceeds the elastic modulus at high frequencies (Figure 5) [11].

**Figure 6:**
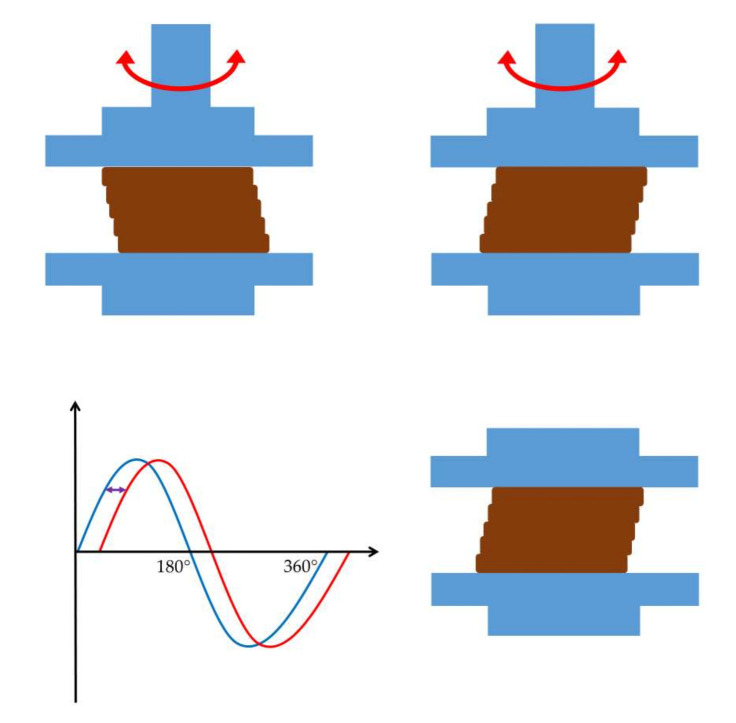
Behavior of elastic materials under oscillational rheological measurements. The graph shows a relatively small-time lag between the blue sine curve of shear strain and the red sine curve of resulting shear stress.

**Figure 7:**
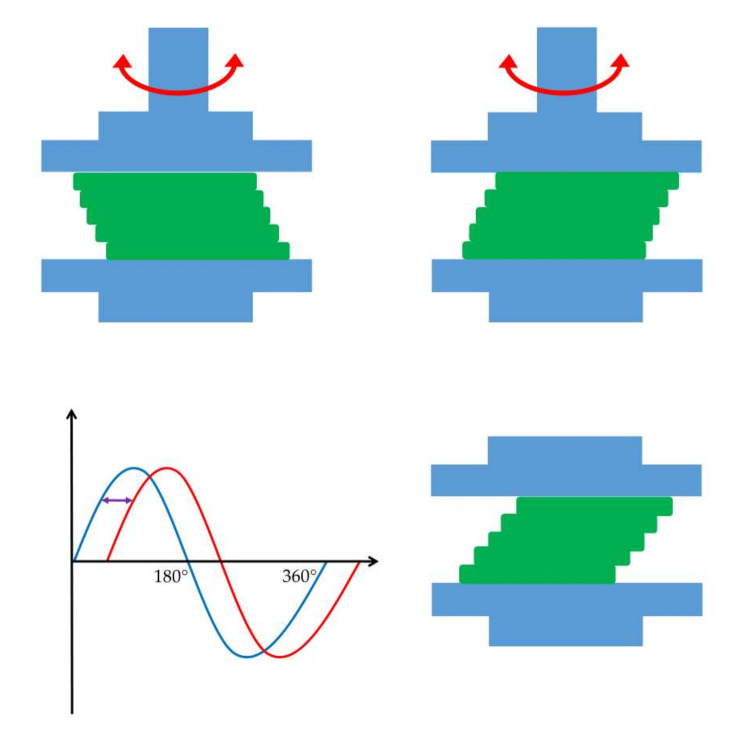
Behavior of viscous materials under oscillatory rheological measurements. The graph shows a relatively large time lag between the blue sine curve of shear strain and the red sine curve of resulting shear stress.

The dominance of the elastic modulus indicates a gel-like material that returns to its original shape after the external force is removed (Figure 6) [13]. On the other hand, the dominance of the viscous modulus expresses the viscous or plastic behavior of the material, which can not regain its original structure when the deforming force ceases (Figure 7) [14].

**Figure 8:**
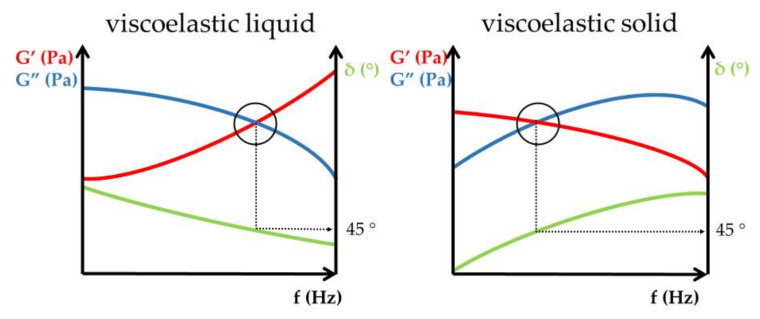
Oscillatory rheology of viscoelastic liquid and viscoelastic solid.

It is worth noting that the phase angle (δ) at the cross-over point coincides with 45°, which is the midpoint of the entire spectrum (0–90°) (Figure 8). A phase angle value of 0° signifies an ideal elastic rheological body and 90° corresponds to an ideal viscous material.

The complex modulus (G*) represents the overall resistance to deformation. The loss factor (tan δ) indicates the ratio of the elastic to the viscous modulus and is defined as G″/G′ (Table 1) [15,16,17].

The determination of the sol–gel transition temperature can easily be performed using dynamic oscillatory experiments. The measurement of the viscous and elastic modulus as a function of temperature clearly and unambiguously assigns the rheological changes associated with the sol–gel transition. The intersection or cross-over point of the moduli (G″ = G′) determines the changes that have occurred in the gel structure and clearly indicates the transition temperature [18,19]. Among the issues of the temperature dependence of mechanical properties, it is worth noting that thermally responsive polymers such as poly(N-isopropylacrylamide) (PNIPAm) or polyvinyl alcohol-based poly(N-isopropylacrylamide) (PVA-PNIPAm) composites undergo a sharp volumetric phase transition around their lower critical solution temperature (LCST), which also affects their viscosity and elastic modulus [20,21,22].

Determining and comparing the above-mentioned parameters can provide valuable information about the rheological properties and gel strength of pharmaceutical and cosmetic preparations [23].

Beyond the oscillatory rheological measurements, new methods for determining viscoelastic properties have already been demonstrated, such as piezotromboelastography, which enables the hemostatic potential of blood to be assessed [24]. Karim et al. published a method for the determination of the viscoelastic properties of samples by tracking the motion of particles by applying molecular dynamics simulations [25].

Ultrasonic spectroscopy is a useful and non-destructive method to characterize the physical and mechanical properties of materials [22,26,27]. Walker et al. published results on a positive correlation between the moduli obtained through ultrasound and mechanical tests applying agarose hydrogels as cartilage phantoms [28]. Nevertheless, smart hydrogels such as poly(N-isopropylacrylamide) (PNIPAm), upon changing their elastic modulus, can have a large impact on the speed of ultrasound, thus greatly influencing biomedical ultrasound imaging [21,29].

**Figure 9:**
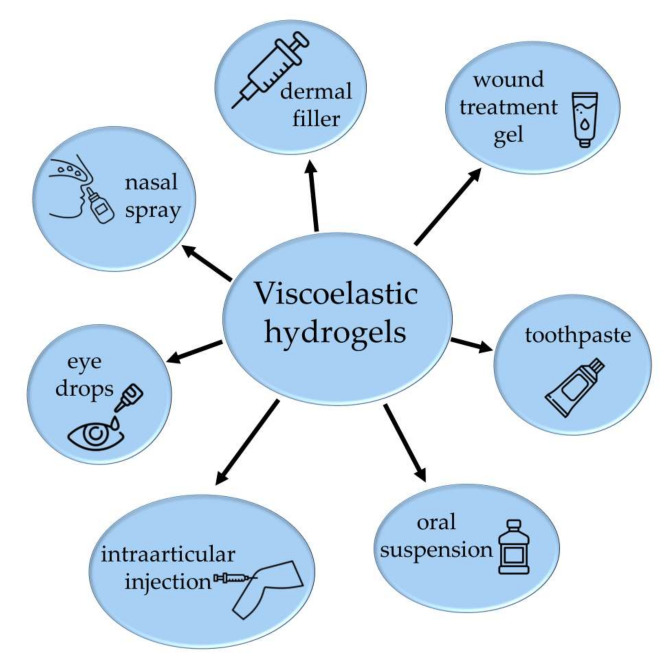
Possible application forms of viscoelastic hydrogels.

The application of viscoelastic gelling agents is intensely spreading due to the additive beneficial properties of the products [30]. Composites of polymers can offer innovative properties with added values in mixtures compared to the application of their individual components. The alteration of the physical properties is strongly dependent on the composition of the hybrid materials and the interactions between guest and host polymers [31]. When measuring the gel strength and modulus of elasticity, it is noteworthy to mention that not only the composition of the hydrogel mixtures but also some additives can have a major impact on the mechanical strength of the gels. Such additives can be divalent ions, which, in the case of biodegradable films, can significantly alter strength and deformation properties [32]. The proper selection of the additives can improve the mechanical properties of the composites [33]. Viscoelastic dosage forms are available in numerous products, not only in intraarticular injections and ophthalmic preparations but also in nasal sprays, oral preparations, wound healing gels and cosmetics on the market (Figure 9) [34,35,36]. Hydrogels with specific unique properties can be used in the areas of targeted drug delivery, regenerative medicine and tissue engineering [37,38,39,40,41].

## 2. Results and Discussion

### 2.1. Hyaluronic Acid (HA)

HA is a linear glycosaminoglycan, a heteropolysaccharide, which is composed of disaccharide units of D-glucuronic acid and N-acetyl-D-glucosamine. By repeating the two building blocks, polymers of different sizes can be created. It can be extracted from cockscombs or obtained biotechnologically through fermentation with bacteria (Streptococcus species). HA is a natural substance that occurs in the human body, in the skin, connective tissue (extracellular matrix), cartilage, synovial fluid, bones and vitreous body of the eye, among other places. It ensures elasticity in the tissues mentioned and has a high water binding capacity [42,43,44,45,46,47,48,49,50,51].

Comparing the viscoelastic properties of healthy and osteoarthritic synovial fluid, a significant loss in molecular weight, HA concentration and elastic modulus is observed in osteoarthritic patients. Healthy young synovial fluid can be characterized by a higher HA molecular weight (6.3–7.6 MDa), higher HA concentration 2.5–4 mg/mL and an elastic modulus of 23 Pa and viscous modulus of 7 Pa at 2.5 Hz. Nevertheless, in the case of osteoarthritic synovial fluid, the molecular weight of HA decreases to 1.6–3.48 MDa, the concentration of HA to 1–2 mg/mL, the elastic modulus to 7 Pa and the viscous modulus to 5 Pa at the same oscillation frequency [47,52].

The study of Rebenda et al. provides insights into the dependence of HA dispersions between rheological and frictional characteristics of articular cartilage. Strong dependency between molecular weight and viscometric, oscillatory rheological behavior was observed. The rheological properties of HA solutions with various molecular weights between 77 kDa and 2010 kDa were analyzed. Higher molecular weight of HA solutions corresponds to higher viscosity and higher dynamic moduli. Concerning the viscoelastic cross-over point determinations, not all samples were found to demonstrate the sol–gel transition in the measured frequency range (0.05–5 Hz at 5% strain). The authors assume that a higher-molecular weight polymer results in a cross-over point of a lower frequency value. No clear dependency between HA molecular weight and frictional properties were determined [53].

Chernos et al. investigated the rheological properties of four novel HA derivates and compared them to the parent HA compound. All derivates were synthesized through deacetylation. One sample was left in its deacetylated form and two others were reacetylated; the final sample was butyrylated. The oscillatory behaviors of all samples were investigated. Although all samples (parent HA and derivates) were found to have viscoelastic properties, a cross-over point of viscoelastic transition could only be observed in the case of the parent compound, the one reacetylated sample and the butyrylated samples. The deacetylation and butyrylation steps are suggested to have an impact on the viscoelasticity of HA samples. This study highlights that the viscoelastic property of HA liquids depends on the method of derivatization, underlining that not just the concentration of HA, average molecular weight and degree of cross-linking can affect the rheological behavior of the polymers [54].

Nicholls et al. investigated the rheological properties of intraarticular hyaluronic acid injections for the treatment of knee osteoarthritis. Their study reveals the complexity of the comparison of the viscoelastic products. Dilute, semidilute and entangled HA injections were compared. The cross-over frequency of the most diluted product was extrapolated and proved to be over 10 Hz. Increasing the hyaluronic acid molecular weight of the injection, the cross-over frequency was shifted to lower frequencies and reached less than 0.01 Hz for the injection with one of the highest molecular weights [55].

Zerbinati et al. studied the impact of polyethylene glycol diglycidil ether (PEGDE) cross linker on the rheological characteristics of hyaluronic acid dermal fillers. The analysis provides insight into the effect of a PEGDE cross linker of the dermal fillers. Non-cross-linked hydrogel showed the lowest elastic property, having the lowest G′, G* and complex viscosity. Elevating the concentration of the cross-linked hyaluronic acid resulted in a direct proportional increase in the elastic properties of hydrogels [56].

La Gatta et al. investigated the rheological properties of BDDE (1,4-butanediol-diglycidylether)-cross-linked hyaluronan hydrogels, which are frequently applied in aesthetic dermatology. It was revealed that the oscillatory rheological properties of the gels were in strong correlation with the amount of cross-linked HA; however, the observed correlation regarding the rate of the overall modified HA was less expressed [57].

The spectrum of HA-containing pharmaceutical preparations is quite wide; they can be used not only in dermal and cosmetic products but also as parenteral in ophthalmic preparations, intraarticular injections and for tissue engineering [42,43,45]. Furthermore, HA and its combinations are intensively researched for tissue engineering.

Hyaluronan-derived products are available on the market for ophthalmic surgery. The first ophthalmic viscosurgical device containing HA was Healon^®^, which was approved in 1980 by the FDA. Since then, several HA-derived eye preparations have reached the market, including eye drops to treat dry eye syndrome [42,43,46].

Numerous dermal fillers are available on the market, such as Restylene^®^, Prelane^®^ and Hylaform^®^ [47,48].

HA can be found in the following marketed viscosupplements: Durolane^®^, Hyalgan^®^, Orthovisc^®^ and Synvisc^®^ [47,49,50,51].

As a summary of HA rheology, it can be concluded that besides the molecular weight, concentration and degree of cross-linking of the polymer, the type of derivatization of HA should be taken into account before the formulation of viscoelastic products.

### 2.2. Methylcellulose (MC)

MC is a partially O-methylated cellulose that is practically insoluble in hot water but forms a colloidal solution in cold water. The gelation of MC is thermoreversible and is attributed to physical cross-linkages of hydrophobic zones.

MC demonstrates a heat-sensitive rheological property that manifests itself in a slight increase in viscosity upon a certain increase in temperature. The anomalous rheological behavior can be explained by the formation of attractive complexes of highly substituted hydrophobic groups within the polymer. The unique thermoresponsive behavior of MC dispersions can be described by the decrease in viscosity up to a critical temperature followed by a particular increase in viscosity as a turbid gel system develops [58,59].

Desbrieres et al. studied the impact of temperature in the range of 20–75 °C on the oscillatory rheological properties of MC solutions. The elastic modulus was initially slightly decreased, and at around 40 °C, an abrupt rise was observed, followed by the development of a turbid gel. Gelation temperature can be defined as the temperature at which the elastic modulus becomes equal to the viscous modulus. Based on the measured data, in the case of an MC solution with a concentration of 39 g/L, the gelation temperature was found to be 51 °C [58].

Due to its beneficial thermoresponsive properties and biocompatibility, MC is a promising polymer matrix for injectable bone substitutes [59]. MC-based injectable systems enable the formulation of bone substitutes that have a chewing gum-like consistency; therefore, they are injectable and form a gel at around 37 °C. The combination of MC with other gelling agents such as gelatin and the addition of bioceramic components affect the mechanical properties and gel strength of the formulations, which should be taken into consideration [59,60].

**Figure 10:**
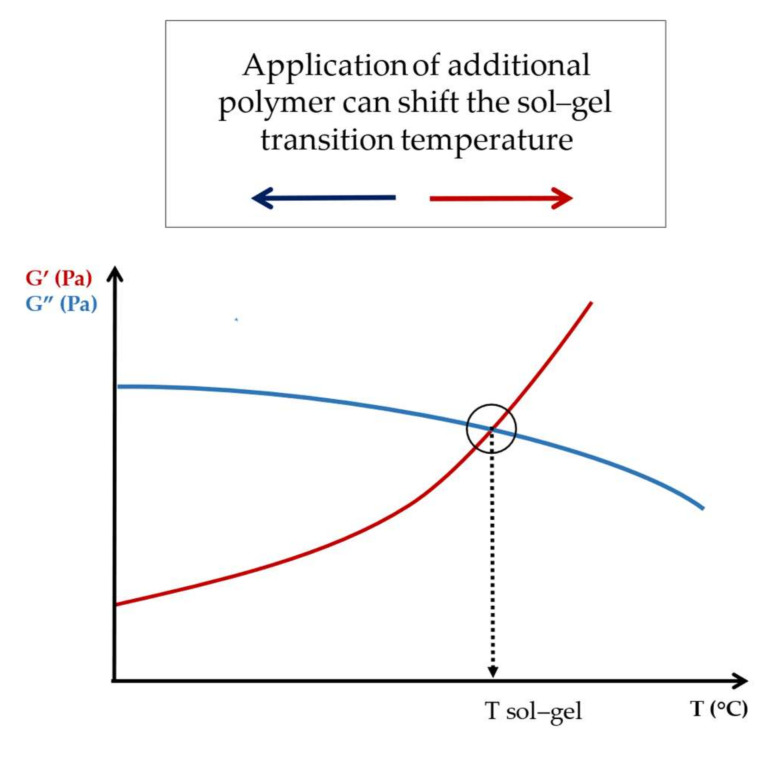
Determination of sol–gel transition temperature using oscillatory rheology.

Liu et al. prepared physical blends of xanthan gum (XG) and MC that proved to be injectable and exhibited a rapid increase in viscosity at body temperature. Among the 8, 10 and 12 wt% MC, the most favorable rheological properties in terms of viscosity and storage modulus were related to the 10 wt% MC. The gelation temperature of the polymer blends can be determined through temperature-dependent oscillation experiments, in which the intersection of the elastic and the viscous moduli indicates the sol–gel transition (Figure 10). After the addition of XG to MC polymers, the gelation temperature was shifted to the lower temperatures, which may be the result of a dehydration process of MC induced by the carboxyl groups of XG [61].

Gupta et al. formulated an injectable intrathecal drug delivery system consisting of a mixture of hyaluronan and methylcellulose (HAMC). Due to the randomly entangled coil structure of HA and the thermoresponsive property of MC, the injectable system has shear-thinning properties before administration and the gel strength increases rapidly at body temperature after injection. The HA affects the gelation temperature of MC similarly to XG, which has carboxyl groups that cause MC to dehydrate, resulting in a decrease in the sol–gel transition temperature [62].

Dynamic oscillatory measurements revealed that MC resembles an elastic gel more, while G′ proved to be higher than G″ in frequency sweep tests at a constant temperature [63].

The combination of methylcellulose with alginate in the presence of calcium ions as physically cross-linking agents makes the gel system an ideal pharmaceutical dosage form for the delivery of heat-sensitive active ingredients. Due to the hydrophobic interactions between the methylcellulose molecules, hydrogen bond formation between -COOH and -OH groups and ionic cross-linking between alginate and calcium ions, the polymer blend demonstrates a network with enhanced physical stability and favorable mechanical properties. MC and alginate hydrogel blends in the presence of calcium ions showed a less significant swelling ratio than polymer blends formulated with NaCl [64].

The synergistic combination of the two polymers results in a gel system with higher viscosity than their individual components. It was revealed that the mixtures have viscoelastic properties with enhanced stiffness. Eskens et al. investigated the rheological effect of epidermal growth factor (EGF) in the MC and alginate mixture. After the incorporation of EGF into the network, a viscosity drop was observed, although an increase in the G′ demonstrated the enhanced effect of elasticity. The thermoresponsiveness of the mixture became more enhanced than in the case of individual components. The authors suggest that hydrophobic interactions between MC and alginate are responsible for the enhanced viscosity and the more expressed thermoresponsiveness of the mixtures [63]. The addition of EGF caused the reduced thermoresponsiveness of the polymer network, assuming that the hydrophilic structure of EGF obstructs the hydrophobic gelling procedure. It is suggested that hydrophobic interactions between MC and alginate cause the more expressed thermoresponsiveness of MC–alginate networks. Increasing the MC concentration, the cross-over point of viscoelastic transition took place at higher frequencies, and a stronger dominance of elastic modulus was observed [63].

The application of polymer blends from MC can open new perspectives in the formulation concepts of thermoresponsive drug delivery systems. The polymer blends of the unique thermosensitive MC and a biodegradable and biocompatible polymer can lead to the formulation of gels with improved physical stability, easier administration and predictable drug release.

### 2.3. Alginate Gels

Alginate is a natural substance that can be obtained from the cell wall of brown algae and various strains of bacteria [65]. Alginic acid is an anionic, hydrophilic polysaccharide consisting of a linear copolymer with homopolymeric blocks of (1 ⟶ 4)-linked β-D-mannuronate (M) and α-L-guluronate (G). The monomers can appear in homopolymeric blocks of consecutive G-residues (G-blocks) or consecutive M-residues (M-blocks). Polymers with a high level of G-blocks form stiff and brittle gels that tend to be more stable than those with high M-block content. In contrast, high M-block content results in soft, elastic gels with high water absorption ability. The mechanical and rheological properties of alginate gels can be adjusted with molecular weight, the concentration of the polymer, cation availability, molecular interactions and G-block distribution [66,67,68].

Based on dynamic oscillatory measurements, the alginate dispersions can be regarded as viscoelastic liquids. The viscous modulus dominates over the elastic modulus in frequency sweep tests, revealing fluid-like viscoelastic behavior [63,69].

Since alginate is a biocompatible polymer with low toxicity, it can be an appropriate carrier for biomedical applications. Alginate hydrogels can be applied for wound healing or tissue regeneration as well. There is great interest in alginate as a coating material for controlled release drug-delivery systems [67,70,71].

In the acidic environment of the stomach, the carboxyl groups of alginate are protonated, providing a high viscosity to the preparations. However, at pH values higher than 3–4, the carboxyl groups become deprotonated, making alginate soluble in the neutral or alkaline conditions of the intestines. Such pH sensitivity promotes the drug release of alginate-based preparations to occur in the intestines [72,73].

Sodium alginate forms a gel foam in the stomach that floats on the contents of the stomach, developing a physical barrier against the hydrochloric acid. Therefore, alginate gels are suitable for the symptomatic treatment of heartburn and acid regurgitation in medicine. An alginate-antacid formulation is available on the market under the name of Gaviscon Double Action liquid^®^. The results of Kwiatek et al. suggest that an alginate antacid raft is formed in the stomach, which eliminates postprandial acidity [74].

Alginate has the ability to form ionic bridges with Ca^2+^ ions above pH 6, developing ionic cross-linkages. Not only Ca^2+^ ions can be applied as cross-linkers, but Ba^2+^ or Zn^2+^ ions as well [67,75].

Cuomo et al. studied the oscillatory rheological behavior of Ca alginate nanodispersion gels. The in situ gelation of 1 wt% sodium alginate was induced by the release of Ca^2+^ from a Ca-EDTA complex at pH 4. It was revealed that the elasticity of the gels is determined mostly by the Ca^2+^ concentrations used as cross-linkers. The highest level of elastic modulus (100 Pa) was reached at the highest Ca^2+^ level when the Ca^2+^ concentration was 10 mM, while in case of low ion levels at a Ca^2+^ concentration of 4–8 mM, just 20 or 50 Pa of G′ were detected. The performed frequency sweep tests revealed the dominant elastic character of the preparations, as in all samples, the elastic modulus exceeded the viscous modulus. The complex viscosity values showed a decrease with increasing frequencies, confirming that the prepared hydrogels have pseudoplastic behavior [76].

The obtained rheological results emphasize that in addition to the molecular weight and concentration of alginate polymer, the cross-linker cation concentration should also be taken into consideration.

### 2.4. Gellan Gum (GG)

GG is an anionic polysaccharide produced by the bacteria Sphingomonas elodea. Two main forms of the polysaccharide are available on the market: high-acyl GG (HAGG) and deacetylated GG or low-acyl GG (LAGG). HAGG contains acetyl and L-glyceryl substituents. Both types of GG can be dissolved in hot water. Decreasing the temperature of GG dispersions results in a gelation by forming thermoreversible hydrogels. However, the GG gels are thermoresponsive, and the thermosensitivity of the gels is over the physiological temperature range, which represents further obstacles in the pharmaceutical development of thermosensitive systems [77].

The effect of different excipients on the rheological parameters of HAGG was investigated by Tako et al. According to their results, intermolecular ionic interactions can lead to an increase in gel strength. CaCl_2_ and urea may contribute to the bonding strength and increase the viscosity of gels. However, the addition of NaOH resulted in a weakening of the gel structure [78]. It was shown by Kasapis et al. that HAGG demonstrates high sensitivity to salts. The viscosity of gels significantly decreased in the presence of NaCl [79].

Dynamic oscillatory measurements were conducted by Osmalek et al. on pure HAGG hydrogels. The results show that the viscous and elastic moduli of temperature sweep tests were nearly constant in the range of 20–40 °C. A strong pH sensitivity of HAGG hydrogels was observed. The acidic pH of simulated gastric fluid (pH = 1.2) caused total destruction of the gel structure. It was suggested that the high concentration of H^+^ ions eliminates the water from the environment of the polymer, which destroys the gel structure. Applying higher pH values (4.5, 7.0, 7.4), the gel structure was retained. It can be assumed that the active ingredients from a pharmaceutical carrier would be released immediately in the stomach due to the low pH values. Gellan gum does not seem to be an appropriate excipient for enteric formulations or dosage forms for prolonged drug release. Oscillatory studies of HAGG and porcine stomach mucin revealed interactions between the two components. It was assumed that after blending the two components, the HAGG liquefies and the mucin precipitates. A significant loss of elasticity was observed in the mixture. The background of the mentioned incompatibility can be explained by the high affinity of mucin for divalent cations such as calcium ions. In the presence of mucin, the polysaccharide gellan gum can not strengthen its gel structure because of the lack of calcium ions, which would form ionic bridges between GG polysaccharide chains [80,81].

It can be predicted that the application of GG hydrogels would lead to similar interaction and incompatibility in mucoadhesive dosage forms such as vaginal preparations. Oscillatory experiments on HAGG and simulated tear fluid revealed that the application of HAGG leads to higher elasticity in the environment of tear fluid than in water. Divalent cations can enhance ionic interactions between the polysaccharide chains of GG, resulting the higher elasticity in the tear fluid containing cations [80].

The results indicate that GG gels are suitable vehicles for ophthalmic preparations, providing prolonged drug release due to the higher elasticity of the formulations. Without the presence of divalent cations, the eye formulations can be instilled in the form of liquid eye drops. The easy administration of low-viscosity eye drops can increase the convenience of application and positively affect patient adherence to the medication.

**Figure 11:**
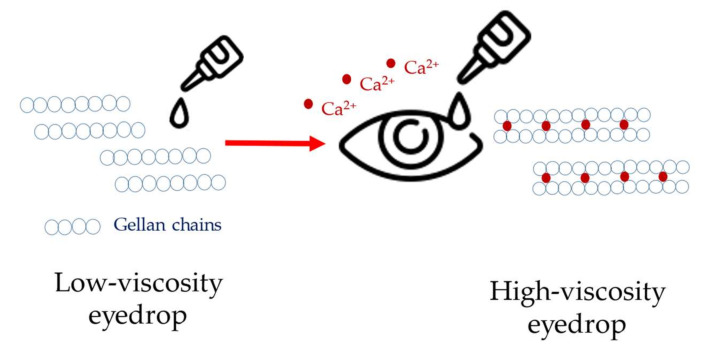
Timoptic^®^ XE gellan gum-containing eye drops with sustained drug release.

After the application of GG liquid eye drops and upon contact with calcium ion-containing tear fluid, the elasticity and, consequently, the viscosity of the GG formulations increases. The higher viscosity is responsible for the prolonged drug release and allows once-a-day administration instead of application two times a day. Timoptic^®^ XE, a Merck marketed product, utilizes the described interaction for timolol maleate in eye drops for prolonged drug delivery (Figure 11) [80,82].

Based on the results obtained, it can be summarized that at low pH values, such as in the stomach or in the absence of calcium ions in the mucins, gellan gum with low viscosity will cause immediate drug release. However, in contrast to these mentioned factors, in ophthalmic preparations, if calcium ions are present, a prolonged drug release will be expected from gellan gum preparations.

### 2.5. Pectin

Pectin is an anionic polysaccharide containing an α-1,4-linked D-galacturonic acid (GalA) unit backbone [83]. Pectin is derived from plants. The major source of the polysaccharide is extracted from citrus peel, followed by apple pomace [84,85,86]. The carboxyl or hydroxyl groups of GalA monomers can be methyl-esterified and/or O-acetyl-esterified. The degree of methyl-esterification (DM) or acetyl-esterification (DA) is used for the classification of pectins available on the market. Low-methoxy pectin (LMP) and high-methoxy pectin (HMP) can be distinguished from each other based on the degree of methyl-esterification. Amidated low-methoxy pectin (ALMP) contains amide groups in its chemical structure [86,87].

Low pH and high sucrose concentration promote the gelation of HMP. The reduced water activity because of the presence of sucrose benefits the chain–chain interactions over chain–solvent interactions. Carboxylate residues are protonated at low pH, reducing the electrostatic repulsion along and between pectin chains. The above-mentioned factors stabilize the HMP gels based on intermolecular hydrogens bonds and hydrophobic bonding between methyl esters [86].

Seshadri et al. described the time-dependent rheological behavior of HMP. A native pectin mixture composed of 1.15% apple pectin and 41.40% sucrose at pH 1.50 was investigated. The oscillatory sweep tests revealed that HMP, an initially viscous gel, became elastic and rubbery. Higher G′ than G″ was detected after 225 min. As time progresses, the pectin molecules build up a network of molecule associations connected by hydrogens bonds and hydrophobic interactions [88].

LMP gels are formed by ionic cross-linkages over a broad pH range. Calcium bridges between two carboxylates derived from different chains known as “shifted egg boxes” promote the gelation [89].

A higher concentration of calcium ions resulted in a more elastic, stronger LMP gel. Additional cross-linking of the GalA backbone leads to a denser gel [90,91].

Mierczynska et al. investigated the effect of various cations on the gelation strength of LMP. It was observed that the addition of calcium or iron ions increased the plasticity of the gels. However, the addition of magnesium ions resulted in a weakened gel structure. It was supposed that iron ions could be replaced calcium ions in the “shifted egg box” model, but there is a lack of information concerning the interactions of magnesium and pectin chains [92].

Löfgren et al. reported that the gel microstructure of ALMP is very similar to LMP. However, ALMP showed reduced sensitivity to calcium ions. It was reported that the low pH generates the gelation of ALMP. The hydrogen bonds between the amide groups promote the acid-induced ALMP gelation [93,94].

The highest viscosity out of the three commercially available pectins was detected in HMP, followed by LMP and ALMP. The results demonstrate that the gel strength is the highest in HMP, indicating that hydrogen bonding in HMP is stronger than the ionic linkages of LMP and ALMP [95].

Pectin-containing hydrocolloids such as CombiDERM^®^ and DuoDERM^®^ can be applied for wound healing [96].

**Figure 12:**
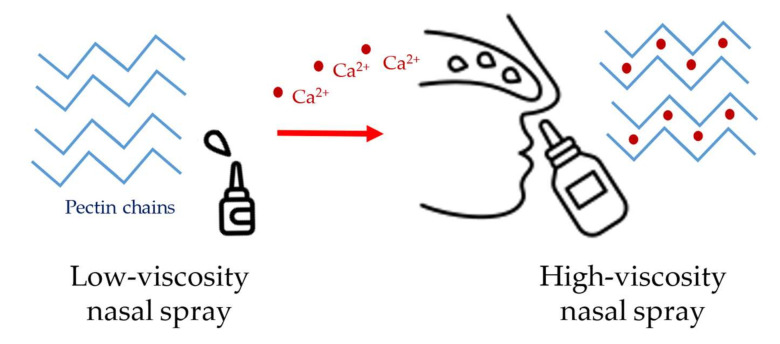
PecFent^®^ pectin-containing nasal spray with sustained drug release.

The ability of pectin to develop Ca^2+^ ion bridges makes it a favorable pharmaceutical carrier in nasal preparations. As calcium ions are present in abundance in the nasal fluid, the gelation of pectin occurs after the application. Due to the presence of calcium ions, the viscosity of the preparation increases. The ionic cross-linking of pectin in nasal spray provides an extended residence time for the formulation. PecFent^®^ is a marketed product that contains fentanyl for the relief of breakthrough pain (Figure 12). The sustained drug release is due to the pectin content, which means that gelation occurs upon contact with the nasal mucus [96,97].

It can be concluded that its calcium binding ability provides pectin with a special property, making it appropriate for sustained drug release upon contact with calcium-containing mucins.

### 2.6. Chitosan

Chitosan is a β-1,4 polymer that consists of linear, unbranched chains of D-glucosamine and N-acetyl-D-glucosamine. It is also known as polyglucosamine. Chitosan is obtained though alkaline deacetylation from chitin, a polymer made from N-acetyl-D-glucosamine extracted from the shells of shrimps, squids and crabs [98,99].

Chitosan has gained a great attention for its diverse applications due to its biodegradability, biocompatibility and non-toxicity [100]. Protonation of the amino acid groups of chitosan takes place below pH 6.2, which makes gelation possible. In aqueous solutions, formic acid or acetic acid can be used for the adjustment of pH [101,102].

It was described that for low chitosan concentrations (0.5–2.5 g/100 g solution), the preparations behaved as pseudoplastic fluids. Measuring the oscillatory parameters of the diluted chitosan solutions, all preparations independent from chitosan and acetic acid concentrations showed no tendency for cross-over points [103].

Richa et al. studied the pH dependence of the gelation of chitosan solutions. Chitosan hydrogels were prepared with acetic acid in a range of 0.05–0.75 M. The highest elastic modulus was measured in chitosan hydrogel synthesized with 0.25 M acetic acid, which indicates the sufficient protonation of chitosan leads to a strongly cross-linked hydrogel. Hydrogels prepared at other molarities showed lower elastic modulus, indicating a lower extent of cross-linking [104].

Tsaih and Chen studied the dependence of the molecular weight of chitosan on its viscosity [105]. Increasing the molecular weight of chitosan generated higher viscosities at the same pH. In addition, a reduction in the viscosity of chitosan preparations was observed during storage, indicating degradation of the polymer [106,107]. An increased deacetylation degree of chitosan corresponded to increased shear viscosity [108]. However, the addition of alcoholic solvents such as ethanol and isopropanol to chitosan hydrogels resulted in decreased viscosity [109].

Martínez-Ruvalcaba et al. reported that the viscosity of chitosan solutions increased when applying more concentrated chitosan solutions. The rising viscosities can be explained by the increased entanglement of macromolecular chains, although the addition of salt or the elevation of the temperature resulted in a decrease in viscosity [110].

Montembaulta et al. searched for the optimal gelation composition of chitosan for the purpose of cartilage regeneration using rheometry. The optimal parameters proved to be the following: 40% degree of acetylation, 1:1 water:alcohol (solvent) ratio and 1.5% polymer concentration [111].

Khunawattanakul et al. investigated the rheological behavior of chitosan and magnesium aluminum silicate (MAS) compositions. It was assumed that the increased viscosity of the blend is due to the electrostatic interactions between chitosan and MAS, as chitosan is positively charged and MAS is negatively charged. It was concluded that chitosan–MAS dispersion offers an effective suspending and gelling agent for pharmaceutical dosage formulations [112].

Nieves et al. prepared chitosan-based hydrogels through ionic cross-linking with propane-1,2,3-tricarboxylic acid (TCA) for sustained drug delivery. The rheological properties of the compounded hydrogels could be modified by altering the chitosan and/or TCA concentrations. According to the measured data, low chitosan concentrations resulted in liquid-like viscoelastic dispersions. In contrast, high chitosan concentrations led to strong gel-like formulations. The viscosity of hydrogels decreased using high TCA concentrations, which caused an increased degree of cross-linking. The study presented the development of a tailor-made sustained release system with controlled rheological properties [113].

Benoso et al. studied the effect of pH in a range of 3.5–6 on the viscosity, viscous and elastic moduli of gelatin and chitosan blends. An increase in pH resulted in increased values of the rheological parameters. In addition, the transition temperatures of gelatin and chitosan blends increased as a function of pH [114].

Bertolo et al. prepared mixtures of chitosan and gelatin, incorporating pomegranate peel extract (PPE) to study the rheological influence of PPE on the hydrogels. The addition of PPE increased the viscous character of the hydrogels. The authors assume that the interaction of phenolic compounds and polymer matrix leads to the formation of a hydrogel with very weak gel strength [115].

Sánchez-Cid et al. investigated the optimal physical properties and preparation conditions of chitosan hydrogels as potential biomaterials to be applied in regenerative medicine. It was found that a pH range from 3.2 to 7 proved to be adequate for the formation of mechanically and morphologically stable chitosan gels, using an agitation time of 1 h, a gelation temperature of 4 °C and a gelling agent concentration of 1.5 wt% [116].

Due to the numerous beneficial properties of chitosan, as it can be characterized as a bioadhesive, bioactive, biodegradable, non-toxic, adsorbable and antimicrobial compound, it is no wonder that the polymer has gained attention in a wide range of biomedical applications [117]. Numerous chitosan-based products can be found on the market for cosmetic purposes, along with various forms of chitosan-based wound dressing materials, such as HemCon^®^ Bandage and ChitoGauze^®^ PRO. Chitosan-containing toothpaste such as Chitodent^®^ is also available on the market to repair tooth enamel [43,72,118].

Before formulating chitosan preparations, not only should the molecular weight of chitosan be considered, but also the preparation conditions, pH and salt and cross-linker concentrations in order to obtain a hydrogel with the required rheological parameters.

### 2.7. Polymethyl Methacrylate (PMMA)

PMMA is a synthetic odorless polymer of acrylic acid. The beneficial properties of PMMA, such as cost-effectiveness and tailorable physical and mechanical properties, make the polymer popular for dental applications and clinical usage. Since its first report in 1843, PMMA has received widespread attention, and its applications in the dentistry field have rapidly expanded, from denture base fabrication to temporary crowns [119].

The PMMA polymer suitable for dentistry should be biocompatible and non-irritating to oral tissues. It should be insoluble in the oral cavity under physiological conditions and have high adhesion to artificial teeth. In addition to beneficial physical and chemical properties, it should possess strong mechanical properties such as a high modulus of elasticity to resist rupture and damage [119,120].

Cheng et al. described the unique characteristics of the rheological properties of PMMA melts. In the polymer melts, the shear shinning phenomenon was observed, and a Newtonian region of the polymer was identified for the low-shear rate regions. The viscosity of polymer melts showing Newtonian flow was greater than 1000 Pa·s, indicating the high resistance to flow of the melts. However, the polymer melts showed non-Newtonian behavior at high shear rates. It was also observed that the viscosity is strongly dependent on the temperature of the polymer melt [121].

### 2.8. Gelatin

Gelatin can be obtained through the hydrolysis of collagen. The physical and mechanical properties, as well as the rheological properties, of gelatin are highly dependent on the animal source and the extraction method used. Gelatin is obtained in large quantities from mammalian sources, but can also be extracted from marine and poultry sources [122].

Gelatin has numerous application fields including the pharmaceutical, cosmetics and food industries [123].

The viscosity of gelatin is influenced by various aspects such as gelatin concentration, pH and temperature. Gelatin exhibits a gel-like structure with a dominant elastic modulus at high frequencies, but its viscous modulus dominates at low frequencies, indicating its liquid-like texture [122,124].

The biodegradability, non-toxicity and easy cross-linking characteristics of gelatin make it possible that the formulation of gelatin hybrid gels may add new innovative properties to the composites in biomedical fields [125].

Hybrid gels made from gelatin and polyvinyl alcohol (PVA), a combination of natural and synthetic biomaterials, offer great potential due to the synergy between the two polymers. Since gelatin hydrogels exhibit low mechanical resistance, thermal instability and a relatively fast rate of degradation, to overcome these disadvantages, gelatin-PVA hybrid gels can be formulated to have improved mechanical properties and bioadhesion, which make them appropriate for wound healing and tissue engineering [31].

## 3. Conclusions and Future Prospects

The application of viscoelastic hydrogels has revolutionized biomedicine. Research into effective biopolymer-containing products is booming today, indicating the tremendous interest in novel hydrogel compositions. The development of viscoelastic pharmaceutical systems is accompanied by numerous marketed products such as viscosupplements, ophthalmic, nasal, injectable preparations and wound dressings. Biopolymers offer a wide range of possible applications in both pharmaceutical and cosmetic developments. Hyaluronic acid, methyl cellulose, alginate, gellan gum, pectin and chitosan are briefly discussed in this review, emphasizing their rheological properties, including viscoelasticity, elastic and viscous moduli.

Based on the articles reviewed in this work, it can be concluded that the selection of the appropriate concentration of the biopolymer is not the only factor that can influence the rheological parameters. The derivatization of the gelling agent, pH adjustment, or appropriate selection of excipients such as calcium ions can also significantly modify the mechanical and physical properties of the products, thereby influencing the rate of drug release and enabling the formulation of sustained-release dosage forms.

It should also be concluded that many various factors and parameters can modify the rheology of the discussed hydrogels. Therefore, each of the gelling agent should be investigated individually based on its particular physical and chemical features and special interactions.

The combination of biopolymers opens up new perspectives in terms of influencing the gel strength or, in special cases, such as the application of methylcellulose mixtures, shifting the gelation temperature and creating injectable pharmaceutical dosage forms. The strength and elasticity of the gel and, thus, the release of the active ingredient can be successfully modified by choosing suitable composition and production parameters.

For the future, it will be essential to thoroughly investigate the beneficial interactions of biopolymer mixtures in terms of their compatibilities, as well as the overall mechanical and physiological parameters of the preparations. In addition to the selection of suitable polymer blends, concentrations, pH adjustment and excipients, the manufacturing processes and application conditions can round off the development process, which leads to the formulation of a physiologically adequate, controlled drug delivery system.

## Figures and Tables

**Table 1:** Rheological parameters of dynamic oscillational measurements.

Parameter	Unit	Description
shear strain (γ)	%	Shear strain triggers the deformation of the rheological body.
Elastic or storage modulus (G′)	Pa	Storage modulus expresses the elastic feature of materials.
Viscous or loss modulus (G″)	Pa	Loss modulus expresses the viscous feature of materials.
frequency (f)	s^−1^	Oscillation frequency of the upper geometry.
phase angle (δ)	° (0–90°)	The difference in sinusoid oscillational curves, which is a measure of the deformation rate.
complex viscosity (η*)	Pa·s	The resistance against flow under oscillational experiments.
complex modulus (G*)	Pa	The normalized value of storage and loss moduli.
loss factor (tan δ)	-	G″/G′; the ratio of the loss (G″) and storage modulus (G′) is the loss tangent (loss factor).

## Data Availability

Not applicable.
